# Damaging effects of UVA, blue light, and infrared radiation: *in vitro* assessment on a reconstructed full-thickness human skin

**DOI:** 10.3389/fmed.2023.1267409

**Published:** 2023-12-01

**Authors:** Paula Montero, Inés Roger, Javier Milara, Julio Cortijo

**Affiliations:** ^1^Department of Pharmacology, Faculty of Medicine, University of Valencia, Valencia, Spain; ^2^Faculty of Health Sciences, Universidad Europea de Valencia, Valencia, Spain; ^3^Biomedical Research Networking Centre on Respiratory Diseases (CIBERES), Health Institute Carlos III, Madrid, Spain; ^4^Pharmacy Unit, University General Hospital Consortium, Valencia, Spain

**Keywords:** full-thickness skin model, photodamage, photoprotection, blue light, ultraviolet-A, infrared light

## Abstract

**Introduction:**

Exposure to solar radiation can cause a range of skin damage, including sunburn, erythema, skin carcinogenesis, the release of reactive oxygen species (ROS), inflammation, DNA damage, and photoaging. Other wavelengths beyond UVB, such as UVA, blue light, and infrared radiation, can also contribute to the harmful effects of solar radiation. Reconstructed full-thickness human skin has the potential to serve as effective predictive in vitro tools for evaluating the effects of solar radiation on the skin. The aim of this work was to evaluate the damaging effects of UVA, blue light, and infrared radiation in a full-thickness skin model in terms of viability, inflammation, photoaging, tissue damage, photocarcinogenesis.

**Methods:**

Full thickness skin models were purchased from Henkel (Phenion FT; Düsseldorf, Germany), and irradiated with increasing doses of UVA, blue light, or infrared radiation. Different endpoints were analyzed on the tissues: Hematoxylin-eosin staining, inflammation mediators, photoaging-related dermal markers and oxidative stress marker GPX1, evaluated by real-time quantitative PCR, as well as photocarcinogenesis markers by Western Blot.

**Results and Discussion:**

The results showed differential responses in cytokine release for each light source. In terms of photoaging biomarkers, collagen, metalloproteinases 1 and 9, elastin, and decorin were modulated by UVA and blue light exposure, while not all these markers were affected by infrared radiation. Furthermore, exposure to UVA and blue light induced loss of fibroblasts and modulation of the photocarcinogenesis markers p53 and p21. In conclusion, the presented results suggest that the various wavelengths of solar light have distinct and differential damaging effects on the skin. Understanding the differential effects of UVA, blue light, and infrared radiation can serve as a valuable tool to investigate the efficacy of photoprotective agents in full thickness skin models.

## 1 Introduction

The skin is the largest organ of the human body, and therefore plays a vital role in safeguarding against various external environmental stressors. Among these stressors, solar light emerges as a primary contributor to skin damage (1). The Radiation that reaches the Earth consists of approximately 9% ultraviolet (UV) radiation, 50% visible light, and 40% infrared (IR) radiation (2). The complete range of UV rays is composed of UVC (100–290 nm), which is absorbed by the ozone layer, UVB (290–320 nm), and UVA (320–400 nm), being UVB the most energetic (3). The spectrum of visible light (VIS) is the responsible for illumination and comprises the wavelengths 400–495 nm (blue light), 495–590 nm (green light) and 590–700 nm (red light) (4). Lastly, infrared (IR) radiation (700 nm to 1 mm) is subdivided into three regions, IRA (700–1,400 nm), IRB (1,400–3,000 nm), and IRC (3,000 nm to 1 mm) (5).

While UVB rays lead to erythema and sunburn (6), UVA rays penetrate deeper into the skin and are responsible for the generation of reactive oxygen species (ROS), dermal damage, and photoaging (7). Blue and infrared light raise more controversy as they are used in dermatological practices, such as the treatment of psoriasis, rosacea and actinic keratosis (8–13). However, recent research confirms that artificial visible light (VIS) induces oxidative stress and tissue damage, causing signs of early photoaging (14, 15). Prolonged exposure to blue light emitted by LED electronic devices is also a factor that increases damage to the skin (4). Specifically, exposure to artificial blue light induces oxidative stress, alters metalloproteinases expression, and triggers the release of inflammatory mediators (4, 16). Regarding infrared light, although the doses used in dermatological practice do not seem to have an effect, vacuolization of epidermal keratinocytes has been observed (13). Additionally, heat is a form of energy that can be transmitted through infrared radiation, leading to an increase in skin temperature (5), induction of inflammatory cytokines and alterations in the production of metalloproteinases like MMP1 (5, 17).

Protection against solar damage is of utmost importance, and at present, the evaluation of photoprotective substances relies on *in vivo* testing methods, specifically the determination of Sun Protection Factor (SPF) and Persistent Pigment Darkening (PPD). However, these approaches are limited in their ability to assess molecular-level protection and the clinical relevance to human health (18). Some authors have evaluated the impact of solar radiation on in-house organotypic models. Their findings demonstrate that these models can react to solar exposure similarly to human skin. This response includes the appearance of sunburn cells, gene expression modulation of dermal markers, and the release of interleukins (19). As a result, the artificial 3D full-thickness skin models have been proposed as alternative approaches to evaluate photoprotection (20). Of note, the European Union Reference Laboratory for the Validation of Alternative Methods to Animal Experimentation (EURL-ECVAM) is actively evaluating these models to validate *in vitro* alternatives (21). However, there are currently no validated assays for assessing photoprotection in 3D skin models.

Understanding the molecular responses to different zones of the solar light spectrum is crucial for deciphering the harmful effects that sunlight can induce on the skin and to develop new photoprotection strategies. While the effects of UVB light have been extensively studied, the mechanisms by which the other wavelengths in the spectrum contribute to solar damage are still not fully elucidated. Hence, the aim of this study is to evaluate the distinct molecular responses of 3D skin models to UVA, blue light, and infrared light. Understanding the molecular relevance of these effects would enable the establishment of evaluation protocols for photoprotective compounds.

## 2 Materials and methods

### 2.1 Cell and tissues culture and treatment

Normal human epidermal keratinocytes (NHEK) (C-12005, PromoCell, Germany) NHEK were cultured in keratinocyte growth medium-2 (KGM-2), supplemented with SupplementMix and CaCl_2_ (60 μM) (Promocell, Germany). Reconstructed human skin Phenion^®^ full-thickness (FT) was produced by Henkel (Dusseldorf, Germany) and cultivated according to supplier instructions using the air-liquid interface medium (ALI-CM, Henkel, Dusseldorf, Germany).

Irradiation procedures were performed in phosphate-buffered saline (PBS). Following irradiation with increasing doses of UVA, blue light (LED) or infrared (IRA), the skin tissues and NHEK cells were immediately transferred to fresh medium and recovered in an incubator (37°C, 5% CO_2_) for 24 h. The UVA and blue light irradiation sources were obtained from Dr. Hönle (Hönle GmbH, Martinsried, Germany). The UVA source consisted of a sun simulator SOL 500, equipped with the H1 filter lens (22). For the preliminary studies in monolayer, the UVA doses chosen were 2.5, 5, 6, 7, 8, 9, 10, 15, and 20 J/cm^2^. For studies in the FT skin models, the UVA doses chosen were 10, 20, 30, and 40 J/cm^2^. The blue light irradiation chamber used was the HONLE LED CUBE 100IC system coupled to the LED Spot 100 HP IC lamp that operates at a constant wavelength of 460 ± 10 nm. For the preliminary studies in monolayer, the blue light (LED) doses chosen were 10, 15, 20, 25, 30, 35, 40, 50, 100, and 150 J/cm^2^. For studies in the FT skin models, the blue light (LED) doses chosen were 40, 60, 80, and 100 J/cm^2^. In both cases, UVA and LED doses were measured using the UVmeter basic radiometer from Hönle. Irradiation with infrared light (IRA) was carried out with the Hydrosun 575 home equipment (Hydrosun Medizintechnik GmbH, Mullheim, Germany), which reproduces deep-penetrating infrared light with wavelengths ranging from 780 to 1,400 nm and an irradiance of 200 mW/cm^2^. The IRA radiation doses for the preliminary studies in monolayer ranged between 60 and 720 J/cm^2^ applied in 5–60 min. For studies in the FT skin models, the IRA radiation doses of 540 and 1,080 J/cm^2^ were applied in 45 and 90 min from a distance of 15 cm. IRA output was determined with a Hydrosun HBM1 (Hydrosun Medizintechnik GmbH, Mullheim, Germany). To control the temperature in NHEK cells, an immersion thermal probe was submerged in the exposed wells. For the 3D skin tissues, the temperature probe was inserted into the dermoepidermal junction. Temperatures were recorded every 5 min.

### 2.2 Cytotoxicity testing

Twenty four hours after irradiation, cell viability was determined using the MTT assay. 1 mg/mL MTT solution was incubated for 3 h at 37°C for both NHEK cells and 3D skin models. After incubation, washes were performed with PBS. To dissolve the formazan precipitate, dimethyl sulfoxide (DMSO) was added for 10 min in the NHEK cells or 2 h in the 3D skin models. Absorbance was measured at 572 nm using the plate reader Spectrostar Omega (BMG LabTech, Biogen, Madrid, Spain). Data were normalized to control values. The cytotoxicity assay was also carried out by measuring lactate dehydrogenase (LDH) release in the medium, using the commercially available LDH cytotoxicity assay kit (Thermo Fisher Scientific, Madrid, Spain), following the manufacturer’s instructions. Absorbance was measured at 490 nm using the plate reader Spectrostar Omega (BMG LabTech, Biogen, Madrid, Spain). LDH contents were normalized to the maximum LDH release.

### 2.3 Cytokine determination by enzyme linked immunosorbent assay

Twenty four hours after irradiation, culture mediums were collected. IL-8, IL-6, and IL-1α cytokine levels were analyzed using commercially available Quantikine^®^ ELISA kits (R&D Systems, Spain) according to the manufacturer’s protocol. Results are expressed as fold change relative to control.

### 2.4 Histological analysis

Twenty four hours after irradiation, the FT skin models were fixed in 4% buffered formaldehyde solution, dehydrated in an alcohol gradient, and embedded in paraffin for sectioning. 5 μM sections were stained with hematoxylin and eosin (H&E) for histological evaluation.

### 2.5 Gene expression analysis by real time RT-qPCR

Twenty four hours after irradiation, the 3D skin tissues were harvested and total RNA was extracted using the Total RNA Purification Kit (Norgen Biotek, Ontario, Canada) following the manufacturer’s instructions. Reverse transcription was performed in 500 ng of total RNA with the Takara PrimeScript RT Reagent kit (Takarabio, Shiga, Japan). The obtained cDNA was amplified with primers and probes predesigned by Applied Biosystems in a QuantStudio™ 5 Real-Time PCR System, using universal master mix (Applied Biosystems, Thermo Fisher Scientific, Madrid, Spain.). The probes used were collagen type I alpha 1 (COL1A1, Hs00164004_m1), collagen type 7 alpha 1 (COL7A1, Hs00164310_m1), decorin (DCN, Hs00754870_s1), elastin (ELN, Hs00355783_m1), metalloproteinase 1 (MMP1, Hs00899658_m1), metalloproteinase 9 (MMP9, Hs00234579_m1) and glutathione peroxidase 1 (GPX1, Hs00829989_gH). Expression of the target gene was expressed as the fold change relative to β-actin (Hs01060665_g1) expression as endogenous control. The mean value of the replicates for each sample was expressed as the cycle threshold (Ct) and the gene expression level was calculated as the difference (ΔCt) between the target gene Ct value and the β-actin Ct value. The fold changes in the target gene mRNA levels were designated as 2^- ΔΔCt^.

### 2.6 Western blotting analysis

Twenty four hours after irradiation the protein content from the 3D skin tissues was quantified using the BCA Protein Assay Kit (Thermo Fisher Scientific, Madrid, Spain). Twenty microgram of denatured proteins were loaded into Mini-PROTEAN^®^ polyacrylamide gels TGX™ (Bio-Rad, United Kingdom), by application of 150 V for 1 h. Proteins were transferred to a nitrocellulose membrane Trans-Blot^®^ Turbo ™ Transfer Pack, using the Trans-Blot^®^ Turbo ™ Transfer System (Bio-Rad Laboratories; United Kingdom). Then, membranes were incubated with 5% bovine serum albumin (BSA) for 2 h and labeled overnight at 4°C with the antibodies p21 (NB100-1941, Novus Biologicals) and p53 (18,032, Cell Signaling). Signal visualization of proteins was carried out by incubating the membranes with chemiluminescence reagents (ECL Plus; Amersham GE Healthcare, United Kingdom). Densitometry of films was performed using the Image J 1.42q software. Results of target protein expression are expressed as the percentage of the densitometry of the endogenous control β-actin.

### 2.7 Statistical analyses

Data are presented as scatter dot blot of *n* = 3 independent experiments run in triplicate, with median and interquartile range values. Normal distribution was confirmed by the Kolmogorov–Smirnov test. Statistical analysis was carried out by multiple comparisons analysis of variance (ANOVA) followed by the Bonferroni *post-hoc* test. *p* < 0.05 was considered statistically significant.

## 3 Results

### 3.1 Evaluation of the cytotoxic impact of UVA, blue light, and IRA irradiation on NHEK cells

**Figure 1:**
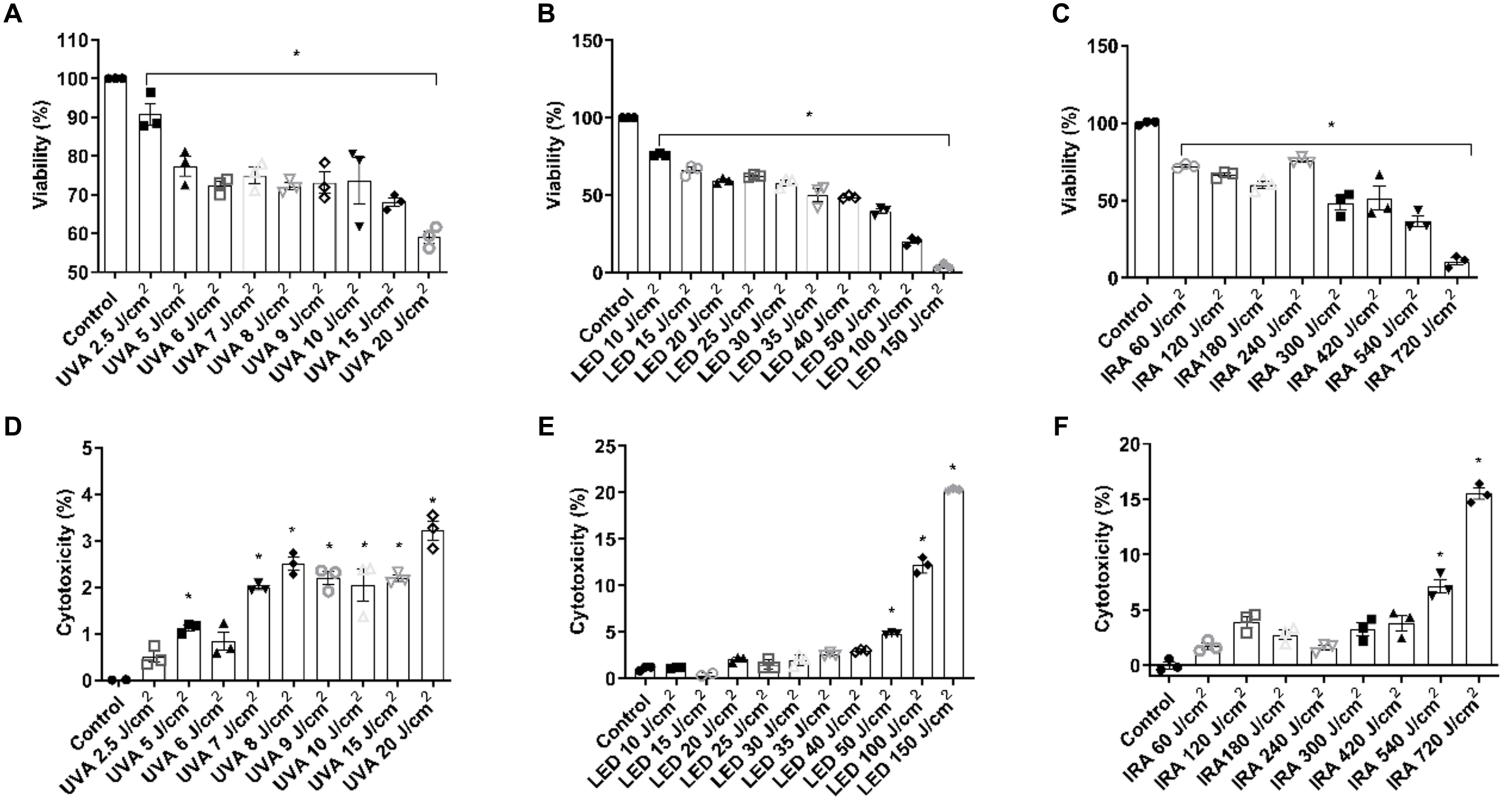
UVA, blue light, and IRA induce the reduction in cell viability and increase in LDH release in NHEK cells. NHEK cells were irradiated with increasing doses of UVA, blue light (LED) or Infrared radiation (IRA). **(A–C)** Twenty four hours after irradiation, the MTT assay was performed. **(D–F)** Twenty four hours after irradiation, the LDH cytotoxicity assay was performed. Data are presented as scatter dot blot of *n* = 3 independent experiments run in triplicate, with median and interquartile range values. Multiple comparisons analysis of variance (ANOVA) was followed by the *post hoc* Bonferroni test. *p < 0.05 vs. control.

To evaluate the range of doses to use in the FT skin models, preliminary experiments were conducted in NHEK cells. Cells were irradiated with UVA at doses ranging from 2.5 to 20 J/cm^2^, and a significant decrease in viability was achieved from 5 J/cm^2^ (Figure 1A). Such reduction was accompanied by an increase in LDH release (Figure 1D). To irradiate with blue light, higher doses, ranging from 10 to 150 J/cm^2^ were used. NHEK viability was progressively reduced reaching 50% at the dose 35 J/cm^2^ (Figure 1B). However, cytotoxicity increased significantly only at the doses of 50, 100, and 150 J/cm^2^ (Figure 1E). Regarding IRA light, the cells were irradiated at doses ranging from 60 to 720 J/cm^2^. Cell viability progressively and significantly decreased, reaching a viability percentage of 10.61 ± 4.3% at 720 J/cm^2^ (Figure 1C). However, cytotoxicity only increased significantly at 540 and 720 J/cm^2^ (Figure 1F).

### 3.2 Cytotoxic effects of UVA, blue light, and IRA irradiation on the full-thickness skin models

**Figure 2:**
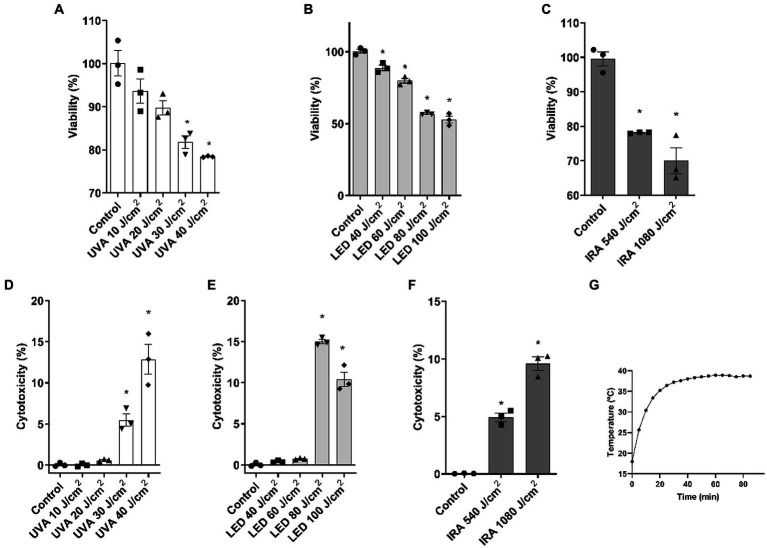
UVA, blue light, and IRA reduce cell viability and increase LDH release in the full-thickness skin model. Full thickness skin models were irradiated with increasing doses of UVA, blue light (LED) or Infrared radiation (IRA). **(A–C)** Twenty four hours after irradiation, the MTT assay was performed. **(D–F)** Twenty four hours after irradiation, the LDH cytotoxicity assay was performed. **(G)** Temperature control at the dermoepidermal junction during IRA radiation. Data are presented as scatter dot blot of *n* = 3 independent experiments run in triplicate, with median and interquartile range values. Multiple comparisons analysis of variance (ANOVA) was followed by the *post hoc* Bonferroni test. *p < 0.05 vs. control.

Based on the cytotoxicity results in cell monolayer, a dosing range was selected for the subsequent experiments in the FT skin models. The ranges used were: 10, 20, 30, and 40 J/cm^2^ for UVA radiation, 40, 60, 80, and 100 J/cm^2^ for blue light, and 540 and 1,080 J/cm^2^ for IRA radiation. As seen in Figure 2A, cell viability was significantly reduced at doses 30 and 40 J/cm^2^. This was correlated with an increase in LDH release (Figure 2D). Exposure to blue light resulted in a significant decrease in viability at all doses (Figure 2B), and, as observed in Figure 2E, cytotoxicity significantly increased at the higher doses of 80 and 100 J/cm^2^. IRA radiation decreased cell viability at both 540 and 1,080 J/cm^2^ doses with a proportional increase in cytotoxicity (Figures 2C,F). For IRA irradiation, the temperature was controlled by inserting a thermal probe at the dermoepidermal junction (Figure 2G). Temperature reached a maximum around 20 min, after which it remained stable, never exceeding 40°C.

**Figure 3:**
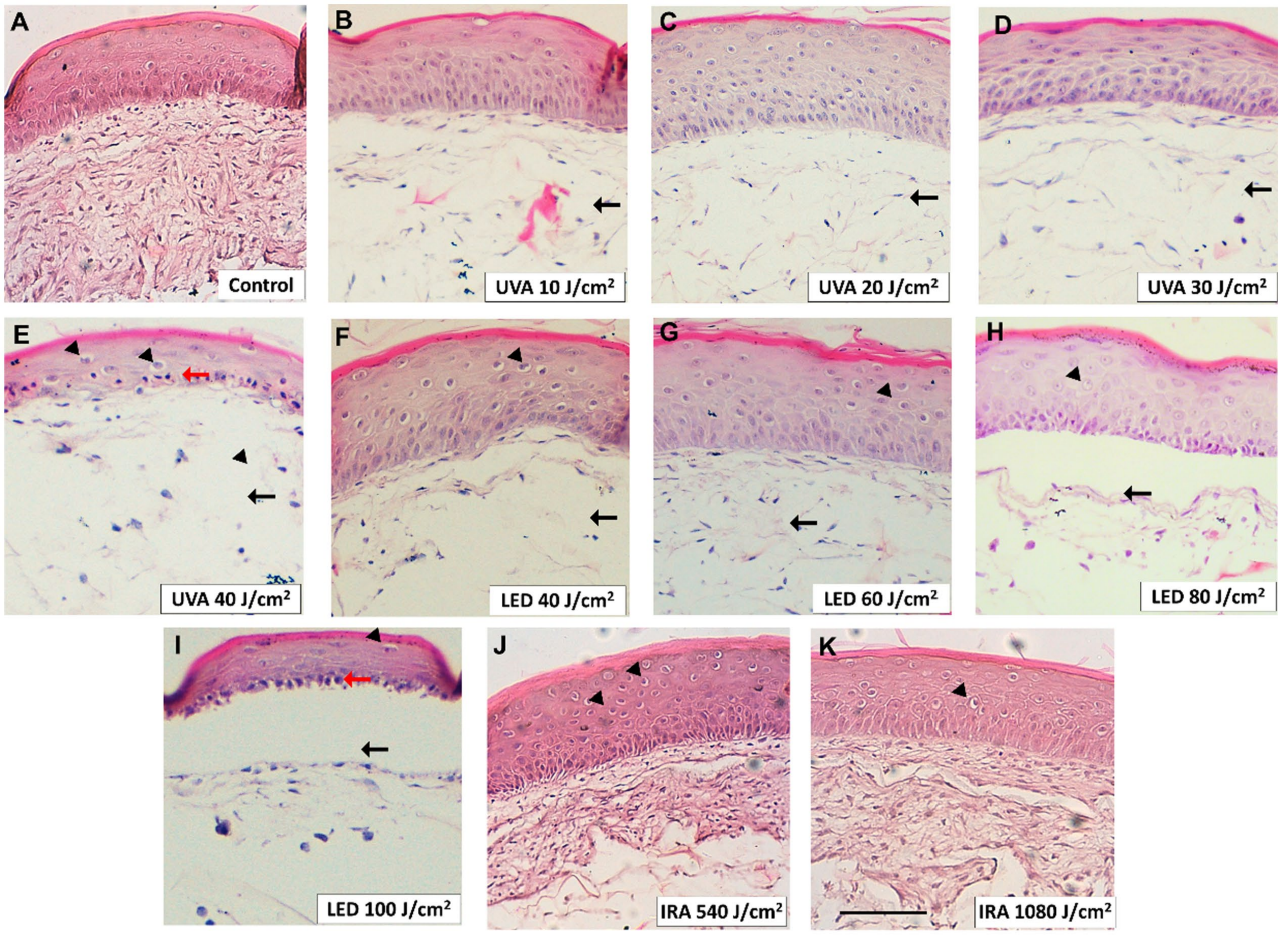
Damaging effects of UVA, blue light, and IRA in the tissue architecture of the full-thickness skin model. Full thickness skin models were irradiated as follows. **(A)** Non-irradiated control. **(B–E)** Increasing doses of UVA light. **(F–I)** Increasing doses of blue light (LED). **(J, K)** Increasing doses of infrared radiation (IRA). Paraffin tissue sections were stained with hematoxylin and eosin. Black arrows indicate loss of dermal fibroblasts. Black arrowheads indicate vacuolization of keratinocytes and red arrows indicate photodamaged keratinocytes with pyknotic nuclei (sunburn cells). Scale bar 100 μM.

The effects on tissue architecture were evaluated using hematoxylin–eosin staining. The control tissue is shown in Figure 3A. UVA irradiation induced the loss of dermal fibroblasts at all doses (black arrows) (Figures 3B–E). Additionally, vacuolization of the epidermal keratinocytes (black arrowheads) appeared at the 40 J/cm^2^ dose. This dose also induced the appearance of pyknotic nuclei in the basal layer of the epidermis (red arrows). Blue light induced morphological changes starting from the dose of 40 J/cm^2,^ including the loss of dermal fibroblasts (black arrows) and vacuolization of keratinocytes in the epidermis (black arrowheads). At the dose of 100 J/cm^2^, the death of the epidermal keratinocytes was visible, with their accumulation in the basal layer and showing pyknotic nuclei (red arrows) (Figures 3F–I). On the other hand, infrared light did not visibly damage the structure of the dermis or the epidermis unlike the other light sources. The only notable morphological effects were the vacuolization of keratinocytes from the granular layer to the basal layer and a slight loss of fibroblasts (Figures 3J,K).

### 3.3 UVA, blue light, and IRA irradiation induce the release of inflammatory cytokines

**Figure 4:**
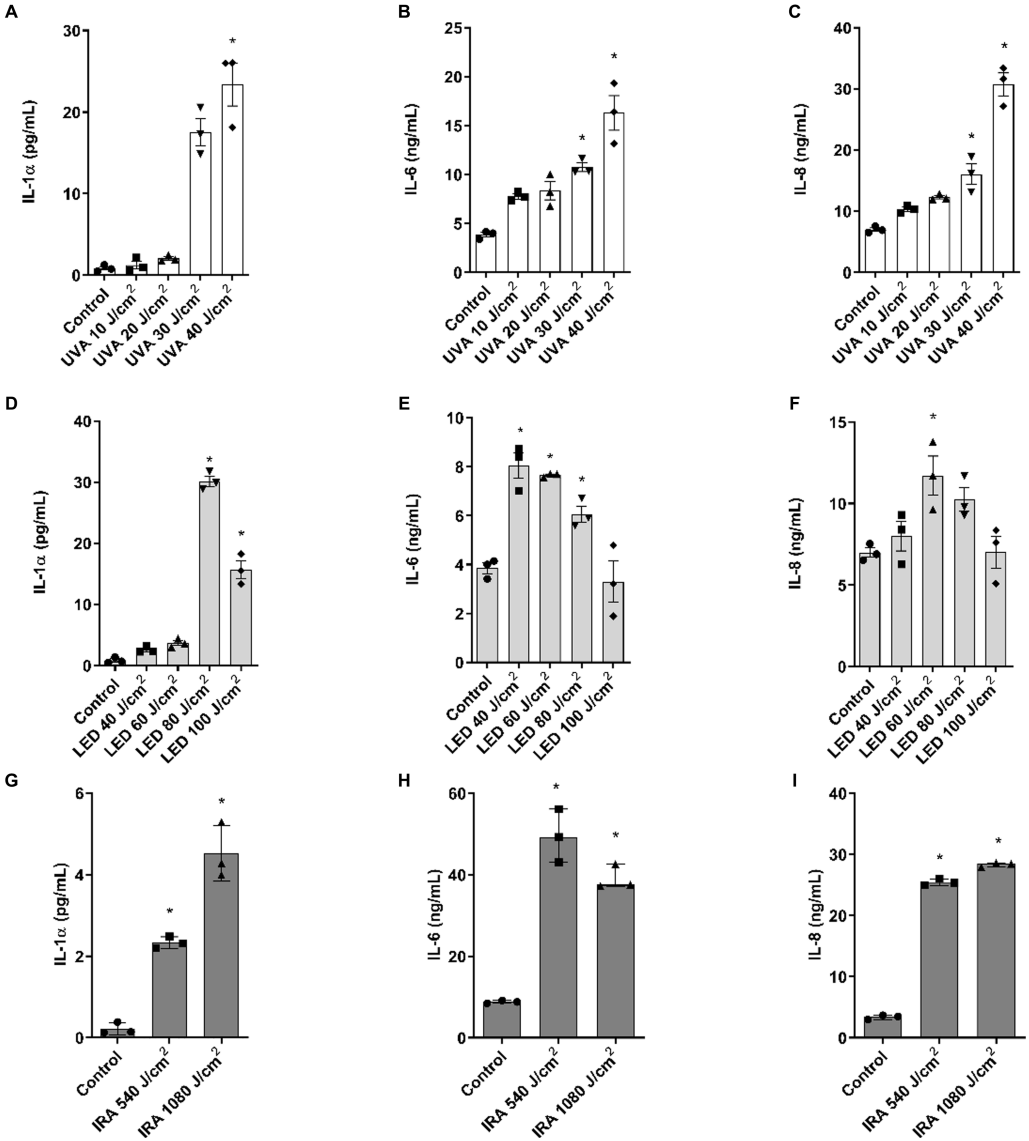
IL-1α, IL-6, and IL-8 release after irradiation with UVA, blue light, and IRA in the full-thickness skin model. Full thickness skin models were irradiated with increasing doses of UVA, blue light (LED) or Infrared radiation (IRA). **(A–I)** Twenty four hours after irradiation, IL-1α, IL-6, and IL-8 levels were measured by ELISA. Data are presented as scatter dot blot of *n* = 3 independent experiments run in triplicate, with median and interquartile range values. Multiple comparisons analysis of variance (ANOVA) was followed by the *post hoc* Bonferroni test. *p < 0.05 vs. control.

Assessment of the released inflammatory cytokines in the culture medium was performed by ELISA. UVA and blue light induced an increase in IL-1α at the highest doses of 30–40 and 80–100 J/cm^2^, respectively, in the range of 20 pg./mL (Figures 4A,D). In contrast, IRA radiation induced a significant increase in the release of this cytokine, with lower levels ranging around 5 pg./mL (Figure 4G). UVA radiation also induced a progressive increase in the release of IL-6 and IL-8, with significant increases observed at the two highest doses (Figures 4B,C). Regarding blue light, IL-6 and IL-8 increased at lower doses of 40 J/cm^2^ and 60 J/cm^2^, beyond which the production of these cytokines started to decrease, creating a bell-shaped histogram (Figures 4E,F). Interestingly, the highest amounts of IL-6 and IL-8 were induced after exposure to IRA radiation (Figures 4H,I).

### 3.4 UVA, blue light, and IRA irradiation modulate the expression of photoaging biomarkers the full-thickness skin models

**Figure 5:**
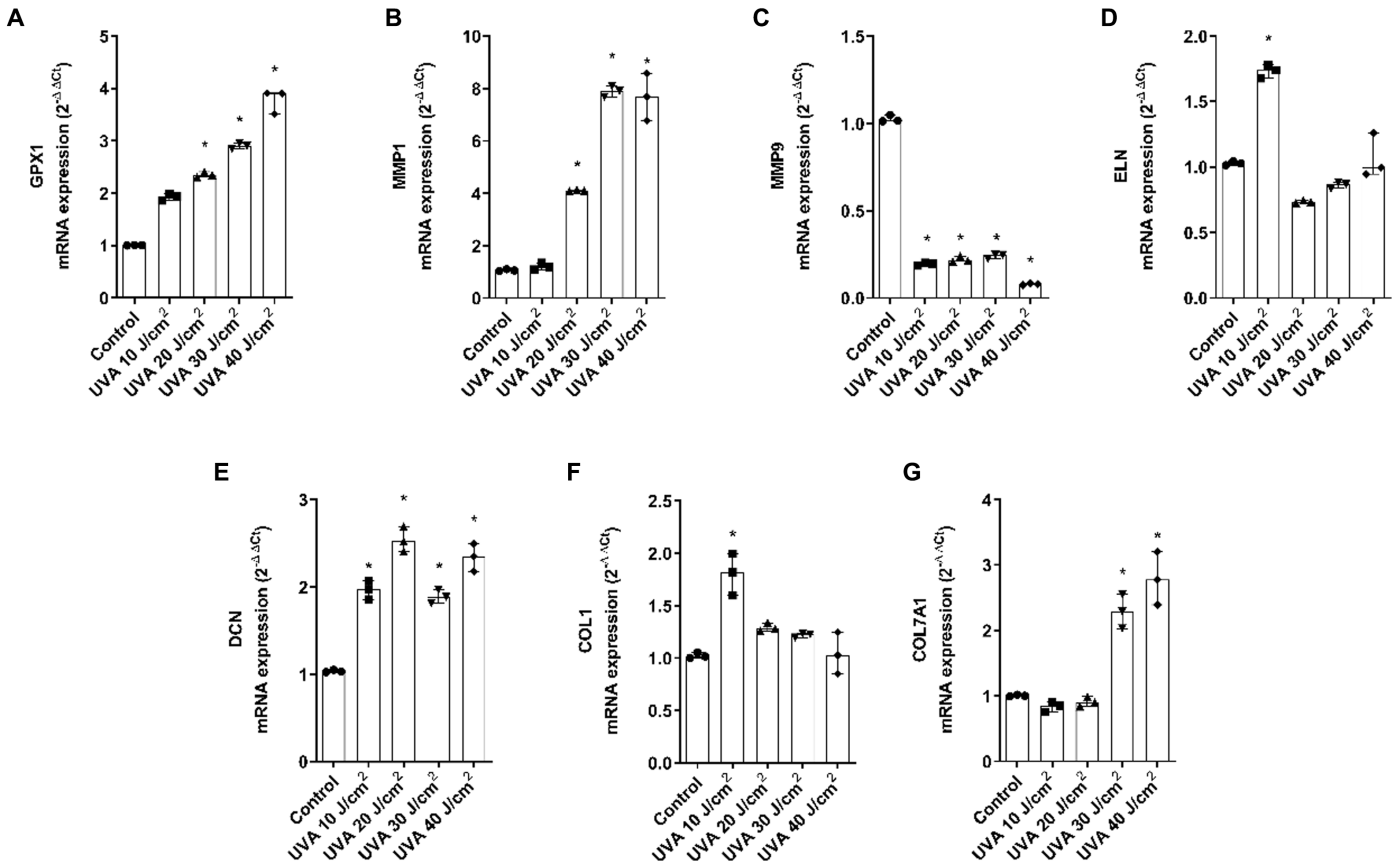
UVA radiation modulates the gene expression of dermal and epidermal markers in the full thickness skin models. Full thickness skin models were irradiated with increasing doses of UVA. **(A–G)** Twenty four hours after irradiation, glutathione peroxidase 1 (GPX1), metalloproteinase 1 (MMP1), metalloproteinase 9 (MMP9), elastin (ELN), decorin (DCN), collagen type I alpha 1 (COL1A1) and collagen type 7 alpha 1 (COL7A1) mRNA levels were measured by real-time PCR. Data are expressed as 2^−ΔΔCt^. Data are presented as scatter dot blot of *n* = 3 independent experiments run in triplicate, with median and interquartile range values. Multiple comparisons analysis of variance (ANOVA) was followed by the *post hoc* Bonferroni test. *p < 0.05 vs. control.

Gene expression analysis was performed by PCR to evaluate the modulation of photoaging dermal biomarkers. As shown in Figures 5A–G, when the FT tissues were exposed to UVA light, the markers GPX1, MMP1, DCN, and COL7A1 were upregulated while MMP9 was downregulated. COL1 and ELN showed similar responses, with an increase at dose 10 J/cm^2^.

**Figure 6:**
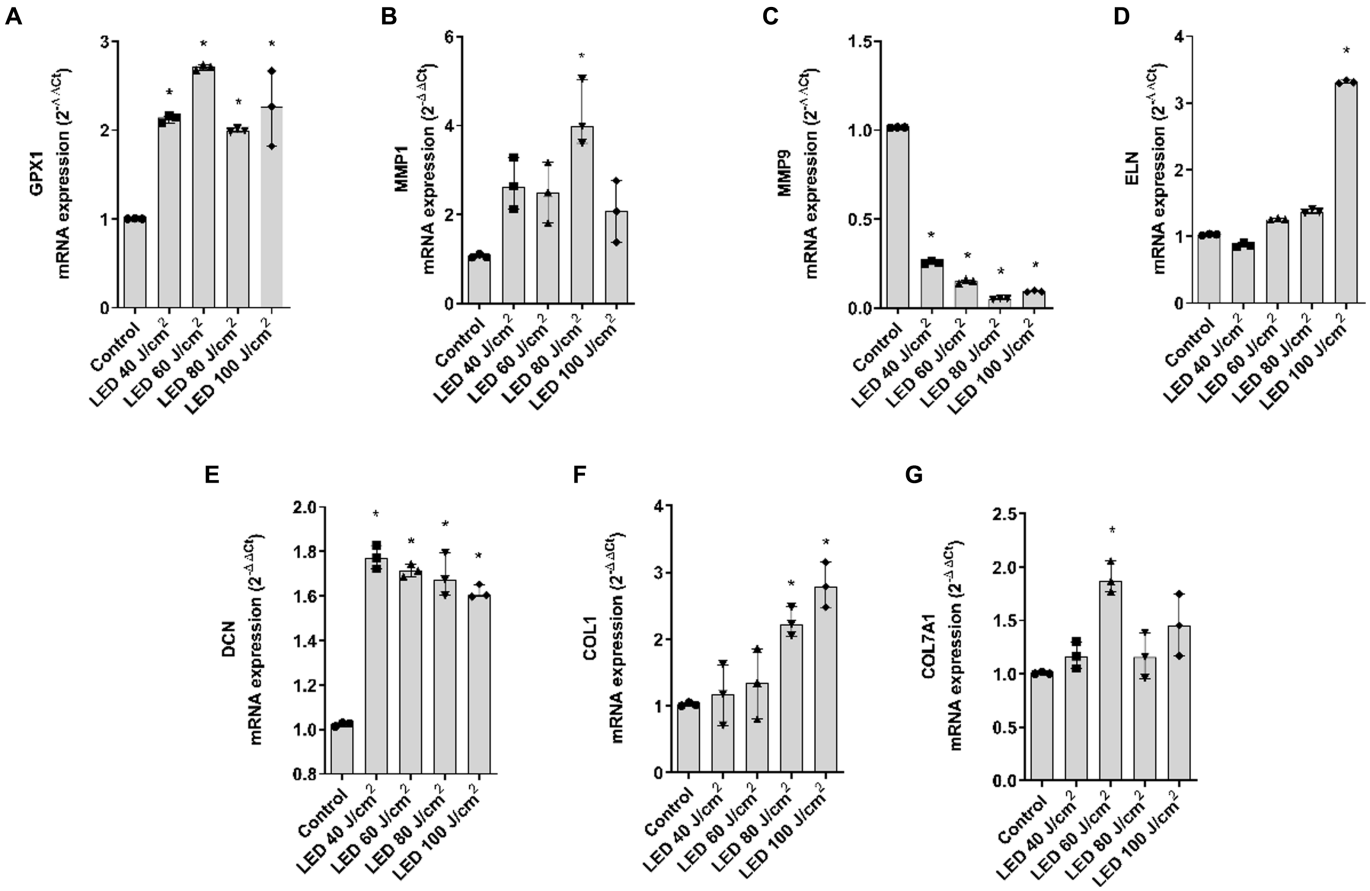
Blue light modulates gene expression of dermal and epidermal markers in the full thickness skin models. Full thickness skin models were irradiated with increasing doses of blue light (LED). **(A–G)** Twenty four hours after irradiation, glutathione peroxidase 1 (GPX1), metalloproteinase 1 (MMP1), metalloproteinase 9 (MMP9), elastin (ELN), decorin (DCN), collagen type I alpha 1 (COL1A1) and collagen type 7 alpha 1 (COL7A1) mRNA levels were measured by real-time PCR. Data are expressed as 2^−ΔΔCt^. Data are presented as scatter dot blot of *n* = 3 independent experiments run in triplicate, with median and interquartile range values. Multiple comparisons analysis of variance (ANOVA) was followed by the *post hoc* Bonferroni test. *p < 0.05 vs. control.

Regarding blue light, GPX1 was significantly upregulated, MMP1 was significantly increased at 80 J/cm^2^, MMP9 decreased after all doses and ELN only increased after 100 J/cm^2^ exposure. DCN, COL1A1, and COL7A1 were also upregulated. While DCN was significant at all doses, COL1A1 was significant at 80 and 100 J/cm^2^ and COL7A1 was significant at the 60 J/cm^2^ dose (Figures 6A–G).

**Figure 7:**
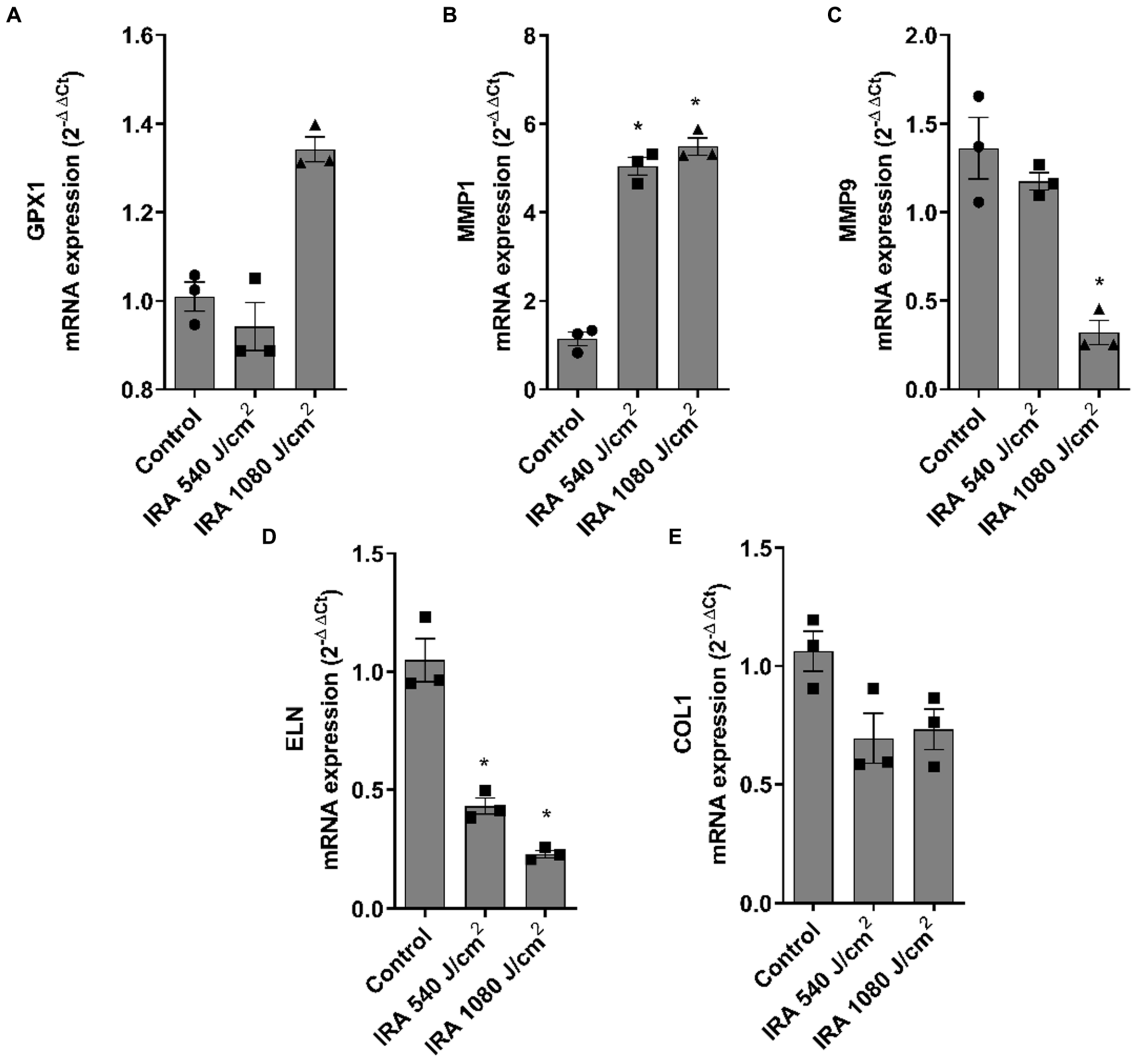
Infrared radiation modulates gene expression of dermal and epidermal markers in the full thickness skin models. Full thickness skin models were irradiated with 540 and 1,080 J/cm^2^ of infrared radiation (IRA). **(A–E)** Twenty four hours after irradiation, glutathione peroxidase 1 (GPX1), metalloproteinase 1 (MMP1), metalloproteinase 9 (MMP9), elastin (ELN) and collagen type I alpha 1 (COL1A1) mRNA levels were measured by real-time PCR. Data are expressed as 2^−ΔΔCt^. Data are presented as scatter dot blot of *n* = 3 independent experiments run in triplicate, with median and interquartile range values. Multiple comparisons analysis of variance (ANOVA) was followed by the *post hoc* Bonferroni test. *p < 0.05 vs. control.

Figures 7A–E show the modulation of the gene markers after exposure to IRA. MMP1 was significantly upregulated and GPX1 showed a non-significant tendency to increase at 1,080 J/cm^2^. Contrarily, MMP9, ELN, and COL1A1 were significantly downregulated at 1,080 J/cm^2^. DCN and COL7A1 were also evaluated, however, IRA radiation did not induce significant changes (data not shown).

### 3.5 UVA, blue light, and IRA irradiation modulate the expression of photocarcinogenesis biomarkers in the full-thickness skin models

**Figure 8:**
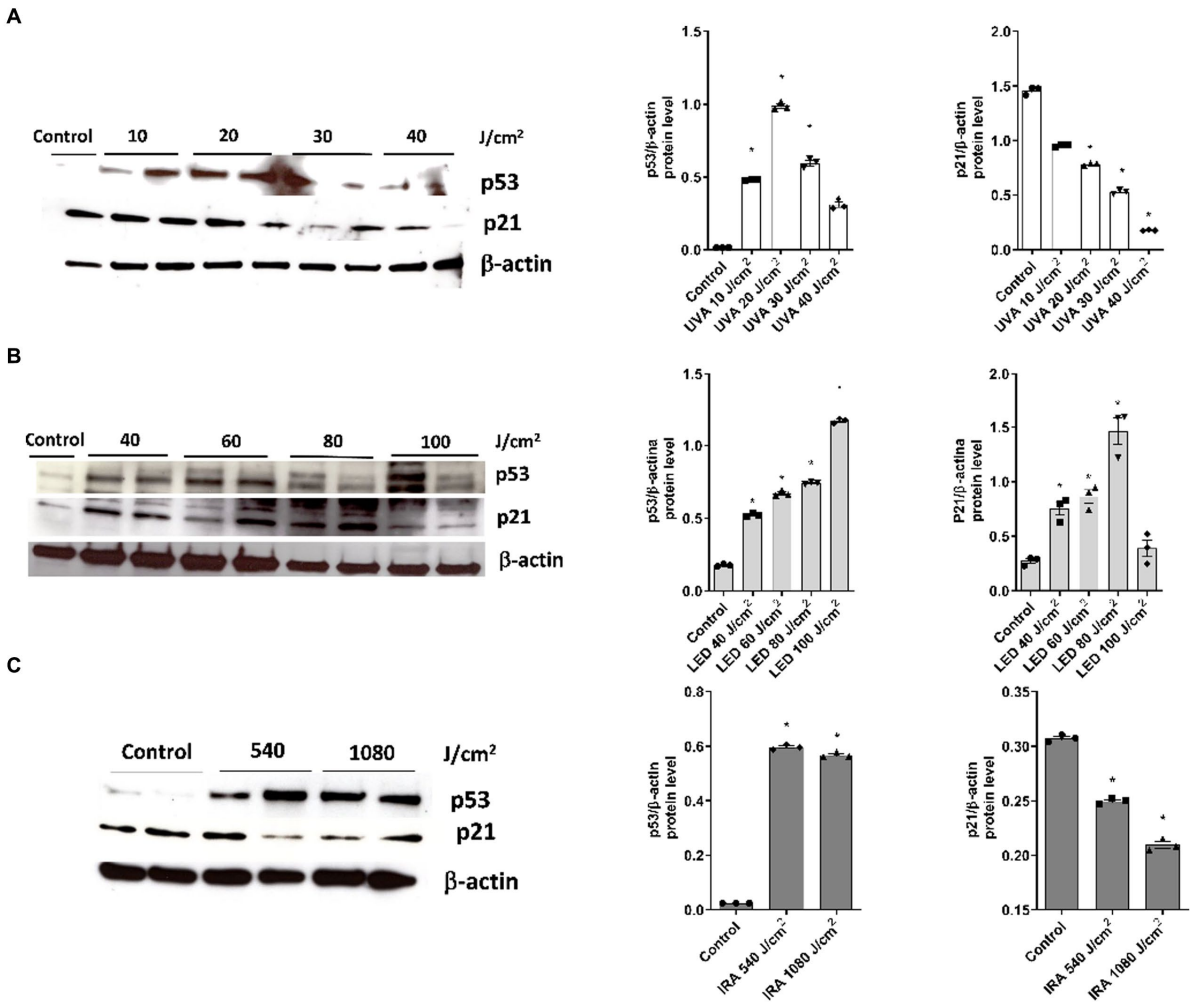
Modulation of p53 and p21 protein expression after exposure to UVA, blue light, and IRA. Full thickness skin models were irradiated with increasing doses of UVA, blue light (LED) or Infrared radiation (IRA). **(A–C)** Twenty four hours after irradiation, p21 and p53 levels were analyzed by Western blotting. Quantification was performed by densitometry and normalized to β-actin. Data are presented as scatter dot blot of *n* = 3 independent experiments, with median and interquartile range values. Multiple comparisons analysis of variance (ANOVA) was followed by the *post hoc* Bonferroni test. *p < 0.05 vs. control.

Western Blot analysis was performed to evaluate the variations in protein expression of p21 and p53. After exposing the FT skin models to increasing UVA radiation, p53 protein expression increased up to 20 J/cm^2^ and then decreased, showing a bell-shaped histogram. p21, on the other hand, exhibited a progressive decrease in protein expression at all doses (Figure 8A). Blue light also significantly induced overexpression of p53, while p21 was increased up to a dose of 80 J/cm^2^. However, at the 100 J/cm^2^ dose, the expression decreased to the level of the non-irradiated control (Figure 8B). After IRA radiation, p53 gene expression significantly increased at doses 540 and 1,080 J/cm^2^, while p21 expression progressively decreased (Figure 8C).

## 4 Discussion

Exposure of the skin to solar radiation is a factor that contributes to sunburn, carcinogenesis, oxidative stress, inflammation, and premature aging of the skin (23). The deleterious effects of UV radiation on the skin have been described, however, understanding the differential effects of UVA, blue light, and IRA wavelengths on the skin is crucial for developing effective photoprotective strategies and assessing the potential harm caused by solar radiation. Therefore, the aim of this study was to assess the suitability of the FT skin model in identifying reliable molecular markers that can discriminate between the damaging effects of different solar wavelengths for future photoprotection studies.

We first conducted a dose-curve analysis in NHEK cells to evaluate the effective doses in monolayer cultures. We observed that higher doses of blue light were needed to achieve similar viability percentages compared to other light sources. However, UVA, blue light and IRA light reduced viability and increased cytotoxicity in NHEK cells. Based on the above results and considering that 3D skin tissues tend to be more resistant to radiation than monolayer cultures, we established the following doses for subsequent experiments in 3D tissues: 10, 20, 30, and 40 J/cm^2^ for UVA radiation; 40, 60, 80, and 100 J/cm^2^ for LED light; and 540 and 1,080 J/cm^2^ for IRA exposure. These doses progressively reduced viability and induced LDH release. Notably, the 3D skin models exposed to IRA did not exceed a temperature of 40°C due to the complexity of the tissue. It is noteworthy that viability and cytotoxicity results show a consistent pattern, although the values are not always complementary. We hypothesize that this discrepancy may arise due to the sensitivity of the MTT assay in detecting viable cells, including those undergoing apoptosis, whereas LDH release is more indicative of necrosis. Consequently, we recommend conducting both analyses to comprehensively assess the overall cellular response. An analysis of the damage to tissue architecture was also performed. Exposure to UVA light resulted in perinuclear vacuolization of keratinocytes at the highest doses, but the major effect was the loss of fibroblasts due to its deeper penetration into the dermal layers. To observe these effects with blue light, the doses had to be considerably increased. Blue light induced the loss of dermal fibroblasts and vacuolization of cellular cytoplasm in the epidermis. Also, at the 100 J/cm^2^ dose, the death of the epidermal keratinocytes occurred, with their accumulation in the basal layer. However, when irradiated with IRA light, only vacuolization and a partial loss of fibroblasts was observed. Other authors have seen similar responses, in non-commercial 3D models, including the disappearance of dermal fibroblasts after UVA exposure (24). Information regarding blue light is more diverse, as some authors indicate that it does not induce cutaneous damage. However, vacuolization in reconstructed skin tissues following irradiation with blue light has been reported (13, 25). These differences exist because studies reporting the safety of blue light exposure generally use therapeutic doses employed in dermatological treatments, which are low doses with short exposure times, unlike the doses used in this study. Also, the effects observed in the FT tissues when irradiated with IRA light are supported by those obtained in other 3D models, where significant impacts on tissue morphology are not observed. The vacuolization observed, could be attributed to the thermal effects of IRA light on the skin (26).

Further, the IL-1α, IL-6, and IL-8 cytokines were measured to assess the inflammatory response of the models to irradiation. Exposure to UVA light, blue light, and infrared light resulted in an increase in the three evaluated inflammatory cytokines. Although there are no studies describing cytokine release in Phenion^®^-FT tissues after solar, blue, or infrared irradiation, other authors have evaluated these markers in non-commercial reconstructed 3D skin tissues. In those models, exposure to UVA and UVB rays led to upregulation of IL-1α, IL-6, and IL-8 (27). In another reconstructed model with Chinese-origin cells, IL-6 and IL-8 were released after UVA exposure (26), and it has also been shown that IL-8 increases in human skin after UV radiation exposure (28). Regarding blue light, contrarily to our results, one study determined that exposure to blue light did not increase IL-1α levels in the forearms of healthy volunteers, however the doses used were much lower than the doses in our study (29). Also, it is proven that blue light induces IL-1α, IL-6, and IL-8 in NHEK keratinocytes (4). Regarding IRA, only one study determined the release of IL-6 in the commercial 3D skin model Epiderm-FT (30).

Next, a series of epidermal and dermal markers were evaluated to determine their potential association with photoaging induced by different lights. The gene expression levels of the antioxidant glutathione peroxidase 1 (GPX1), the dermal markers decorin (DCN), elastin (ELN), and collagen type 1 (COL1A1), as well as matrix metalloproteinases 1 and 9 (MMP1, MMP9), and the marker of dermis-epidermis junction integrity, collagen type 7 (COL7A1) were measured. GPX1 increased after UVA and blue light exposure, but not significantly after IRA exposure. This increase suggests the activation of the antioxidant defense system (31). Consistently, an increase in reactive oxygen species (ROS) has been reported after *in vivo* UVR irradiation (32) and after exposure to both blue light and IRA in skin fibroblasts *in vitro* (33–35). Matrix metalloproteinases (MMPs) are enzymes responsible for degrading the extracellular matrix (36). Our results showed that all the lights studied induced an increase in MMP1 and a decrease in MMP9. Exposure of reconstructed skin to UVA has been reported to induce the production of MMP1, mainly by dermal fibroblasts (37, 38), contributing to the degradation of collagen and elastin fibers during the photoaging process (39). Studies also indicate that human fibroblasts increase the expression of MMP1 after exposure to IRA light, independent of its thermal effects (40, 41), and the same occurs in humans, but with considerable interindividual variability (42). Further, an increase in MMP1 has been shown in keratinocytes exposed to blue light (33), which aligns with our findings. Regarding MMP9, the metalloproteinase responsible for elastin and fibronectin degradation, there is less available information. *In vivo* studies have shown that UVB exposure increases MMP9 expression (43), as observed in reconstructed models such as Epiderm-FT (44). Some authors have reported early downregulation of MMP9 after UVA irradiation, likely due to an inhibitory effect of UVA mediated by singlet oxygen release. This shows an inverse response to UVA irradiation in NHEK cells compared to fibroblasts, which confirms the importance of the use of 3D models in photoprotection studies. Regarding blue and IRA light, there is less information in their influence in modulating MMP9, however, *in vivo* studies describe an increase in MMP9 induced by IRA light (34, 45).

ELN and COL1A1 elicited similar responses. After UVA irradiation, both markers increased their expression at the lower dose and blue light elicited an increase, but at 100 J/cm^2^ dose. On the contrary, after IRA exposure, both markers were decreased. The increase in ELN and COL1A1 after UVA may be a defense mechanism of the tissue to counteract matrix degradation (46). In line with our study, decreases in COL1A1 have been shown after exposure to IRA light in healthy volunteers (30, 40, 47). However, there are also studies reporting an increase in COL1A1 and ELN after low-dose IRA light therapy, although these results are obtained upon exposure to low doses (48). Further, UVA and blue light induced an upregulation of DCN similarly to the results obtained by Meloni et al. after UVA irradiation (46).

Next, we evaluated the markers p53 and p21 as part of the molecular events involved in the development of carcinogenesis. Upon UVA irradiation, p53 expression showed a bell-shaped curve modulation, with a decrease in expression at higher dosages, along with a decrease in p21 expression. This pattern of p53 expression in a bell-shaped curve after UV exposure has been described before in non-commercial skin models (49). Additionally, it has been observed that UV exposure increases p53 in primary keratinocytes (50), while in UVB-irradiated keratinocyte cell line (HaCaT), p53 levels remain unchanged and p21 decreases (51). Conversely, another study in HaCaT cells described negative regulation of p21 and p53 after UVB (52). On the other hand, 3D skin tissues irradiated with blue light showed increased expression of p53 and p21, except at the highest dosage where p21 decreased. In response to IRA radiation, p53 increased and p21 decreased. Of note, the influence of blue and infrared light on carcinogenesis has not been deeply studied. In human skin biopsies, a temporary decrease in p53 expression was observed 24 h after blue light exposure (13). These results suggest that the functions of p53 as “guardian of the tissue” in human skin and the control of apoptosis pathways by p21 can be translated to the FT skin models for their use in photoprotection studies. It should be noted that the response in the expression of these proteins is influenced by both the dosage and the time of endpoint measurements. Therefore, their modulations can vary between different experimental conditions. In general, the increase in p53 and the downregulation of p21 imply that after irreparable damage, apoptosis mechanisms are activated.

Taken together, the results obtained confirm that the FT tissues are a valuable tool for evaluating the different effects produced by the lights of the solar spectrum. Furthermore, these 3D models have confirmed that the less studied lights in the spectrum, blue and infrared light, are also harmful to the skin at high doses. These findings are of vital importance, as continuous exposure to LED light sources from electronic devices, as well as the thermal effect caused by IRA, could cause more damage to the skin than previously believed. The chosen doses in all cases were doses above the minimum biological levels to ensure the molecular response of all evaluated markers. However, it is difficult to extrapolate the actual equivalent doses used in these experiments. For example, UV dose is highly influenced by the source of irradiation, as well as latitude, season, time of day, solar elevation angle, weather conditions, ozone layer thickness, and pollutants (53). According to Ryšavá et al., a dose of 10 J/cm^2^ of UVA is equivalent to an exposure of approximately 1 h of sunlight in May at latitude (49°N) (22). Regarding blue light, according to measurements performed by Rascalou et al., the dose of 90 J/cm^2^ used in this study would be equivalent to an exposure to digital devices for approximately 1,700 h, which is a chronic exposure averaging 6 h per day over 283 days (15).

Also, although these evaluations are promising, more tests comparing ISO standards with commercial formulas with different SPF values must be conducted before establishing photoprotection protocols in these 3D skin models. It is noteworthy that some authors have performed these tests on other reconstructed models and found that a higher SPF does not necessarily translate into greater biological protection. This confirms that available methods, such as SPF or PPD, do not reflect broad-spectrum photoprotection as they do not consider the impact of each type of light. Moreover, when based on erythematous response, SPF does not adequately predict the level of protection against other biological damages (38, 54, 55). Therefore, the need for validated methods to evaluate the protection of cosmetic formulations at the biological level is significant. In conclusion, the FT skin models presented in this work have proven to be a valuable tool for studying the effects of the different lights in the solar spectrum. The findings emphasize the need to be cautious about prolonged exposure to blue light from electronic devices and infrared light and highlight the need for reliable methods to test and validate photoprotective agents.

## Data availability statement

The raw data supporting the conclusions of this article will be made available by the authors, without undue reservation.

## Author contributions

PM: Conceptualization, Data curation, Formal analysis, Investigation, Methodology, Writing – original draft, Writing – review & editing. IR: Investigation, Methodology, Writing – review & editing. JM: Funding acquisition, Resources, Supervision, Validation, Writing – review & editing. JC: Conceptualization, Formal analysis, Funding acquisition, Resources, Supervision, Validation, Writing – review & editing.
